# Large-Scale Sequence Analysis of Hemagglutinin of Influenza A Virus Identifies Conserved Regions Suitable for Targeting an Anti-Viral Response

**DOI:** 10.1371/journal.pone.0009268

**Published:** 2010-02-17

**Authors:** Leepakshi Sahini, Anna Tempczyk-Russell, Ritu Agarwal

**Affiliations:** 1 Department of Process and Analytical Biochemistry, ChimericBio Incorporated, Long Island City, New York, United States of America; 2 Department of Computational Biology and Chemistry, Accelrys Incorporated, San Diego, California, United States of America; University of Minnesota, United States of America

## Abstract

**Background:**

Influenza A viral surface protein, hemagglutinin, is the major target of neutralizing antibody response and hence a main constituent of all vaccine formulations. But due to its marked evolutionary variability, vaccines have to be reformulated so as to include the hemagglutinin protein from the emerging new viral strain. With the constant fear of a pandemic, there is critical need for the development of anti-viral strategies that can provide wider protection against any Influenza A pathogen. An anti-viral approach that is directed against the conserved regions of the hemaggutinin protein has a potential to protect against any current and new Influenza A virus and provide a solution to this ever-present threat to public health.

**Methodology/Principal Findings:**

Influenza A human hemagglutinin protein sequences available in the NCBI database, corresponding to H1, H2, H3 and H5 subtypes, were used to identify highly invariable regions of the protein. Nine such regions were identified and analyzed for structural properties like surface exposure, hydrophilicity and residue type to evaluate their suitability for targeting an anti-peptide antibody/anti-viral response.

**Conclusion/Significance:**

This study has identified nine conserved regions in the hemagglutinin protein, five of which have the structural characteristics suitable for an anti-viral/anti-peptide response. This is a critical step in the design of efficient anti-peptide antibodies as novel anti-viral agents against any Influenza A pathogen. In addition, these anti-peptide antibodies will provide broadly cross-reactive immunological reagents and aid the rapid development of vaccines against new and emerging Influenza A strains.

## Introduction

The recent outbreak of swine-origin influenza A (H1N1) that began in April 2009 in Mexico has caused an immediate international concern. In June 2009, the virus had already spread to 70 countries and a global pandemic was declared by WHO [1]. Since then the virus has continued to spread to 168 countries and has infected approx. 209,438 people worldwide [1]. Over the past decade, influenza epidemics have been mild; nevertheless, influenza A virus has been predicted as a major and unpredictable threat to public health due to historic precedents [2], [3]. Prior to the outbreak of H1N1, H5N1 influenza virus infection in humans in South Asia had caused a significant number of cases of severe disease and deaths in humans and had led to a global concern about the potential of this virus to evolve to pandemic proportions [4]. These current and recurring events of Influenza A fatalities around the world highlight this ever-present threat to global public health.

The inability to provide lasting protection to humans against influenza A virus is due, in part, to the rapid evolution of the viral surface glycoprotein, hemagglutinin (HA), which leads to a change in its antigenic structure. Hemagglutinin plays a major role in determining host specificity since it is responsible for viral binding to host cell receptors and penetration of host membranes [5], [6], [7]. Influenza A hemagglutinin exists as 16 related subtypes in birds [8], [9]. Three subtypes, H1, H2 and H3, are found in viruses known to have caused human pandemics and several subtypes are known to infect other mammals, e.g. pigs and horses. During repeated rounds of infection, selection, and re-infection, influenza viruses undergo host-specific adaptations. The regions involved in host-virus interactions including the receptor-binding site are likely to resist changes, but the antigenic sites are subject to drift due to immune surveillance. In addition, some regions may evolve for other reasons e.g. to facilitate post-translational modification or to facilitate protein folding and maintenance of secondary/tertiary structures [5]. It is reasonable to hypothesize that regions of the hemagglutinin protein that are phylogenetically information rich, would be good candidates for involvement in virus-host interactions and for additional viral functions. This would be especially true for regions shared by more than one subtype. In this work, we attempt to identify such information-rich regions on the HA1 subunit of the HA protein, where the majority of the amino-acid variation is located. This subunit is also highly exposed and, hence, a target of neutralizing antibody responses [10]. Currently, it is not possible to modulate the B-cell response to specific protein regions, and hence, the current vaccines, which are composed mainly of HA protein or inactivated virus, have to be reformulated as the virus mutates and changes. Due to constant evolution of influenza A viruses, there is an urgent need for the development of new vaccine strategies and anti-viral therapies based on conserved regions, which can provide wider protection against any new Influenza A virus.

This study focuses on analysis of Influenza A human HA1 protein sequences available in the NCBI database, corresponding to H1, H2, H3 and H5 subtypes. These sequences were used to identify nine regions that were conserved across subtypes. These conserved sites were further analyzed in terms of secondary structure, hydrophilicity and solvent-accessible surface to determine their suitability for targeting anti-peptide antibodies/anti-viral therapies.

This work will be critical in the development of new anti-viral therapies such as peptide antibodies or a peptide vaccine that will be effective against all current and possibly future Influenza A strains. Further, due to evolutionary variability of the HA protein, development of vaccines against emerging strains is delayed by the non-availability of reagents/antibodies that recognize the new viral strain. This type of information will also be useful in designing broadly cross-reactive, immunological reagents/peptide antibodies to quantify the new vaccine product until viral-specific reagents become available.

## Methods

### Analysis of HA Sequences

All full length HA sequences were downloaded from NCBI Flu database (as of July 2009). Separate files were generated for all H1, H2, H3 and H5 sequences. Computing a simultaneous multiple alignment across all subtypes for the HA segment is challenging for several reasons including: 1) high sequence-variability between subtypes, 2) large number of sequences that need to be aligned and 3) the need to correct for biased representation of the subtypes in the sequence databases. As a surrogate, we aligned the sequences in stages where we used a sensitive profile-profile alignment stage to align sequences across subtypes. In order to achieve this, we first aligned the HA sequences within each subtype using MUSCLE 3.6 [11] after filtering out frame-shifted and partial sequences (based on an initial MUSCLE alignment and manual curation). We also excluded subtypes that were poorly represented in the NCBI database to eliminate any further bias in the results. The profiles for each multiple-alignment were then aligned to the other profiles using the profile-profile aligner COMPASS [12]. Because the profile for H2 had the most significant alignments to the other profiles, as measured by COMPASS E-values, it was used to create a seeded multiple alignment for the profiles similar to a PSI-BLAST [13] alignment. The profile multiple-alignment was then expanded, based on the multiple-alignment for each profile, into a multiple-alignment for all the sequences across subtypes. In the final alignment, the sequence of 1HGJ (H3 HA structure) was included to follow the H3 numbering.

Computing a conservation score for each column of the final multiple-alignment was complicated by the uneven representation of the subtypes in the alignment. Since weighted entropy scores require ad-hoc weighting schemes for the various sequences in the alignment, we instead relied on a simpler correlation-based score: for each of the subtypes H1, H3 and H5, the dot product of the profile column with the corresponding profile column of H2 was used to estimate the correlation of the profiles. The correlation with H2 was chosen because H2 was the seed profile for the multi-alignment. The correlation values were rounded to two decimal places and hence some correlation values were not exactly 1.0 but very close to 1.0. The final conservation score was then taken to be the minimum of these correlation scores, as a conservative estimate of conservation. The above alignment results and their correlation scores are shown in Table S1.

We concentrated on the HA1 subunit and manually identified regions containing 6 residues or more, where more than 50% residues had a final conservation score of 0.9–1.0, and selected them as conserved regions. We chose a threshold of 6 residues as a minimum length required for a peptide antibody response.

### Analysis of HA Structure

All of the structure analysis was done using Accelrys software discovery studio 2.0. The H3 structure (1HGJ) was downloaded from the PDB site. A ribbon model of the HA monomer was generated to depict the nine identified conserved regions. Each region was enlarged to show the detailed three-dimensional (3-D) structure of the above sites. The H1 structure (PDB: 1RVZ) was used for comparison with the H3 structure.

The secondary structure was calculated using both DSSP secondary structure prediction program [14] and from the PDB file. Since the results of the two methods were similar, only the results from DSSP program were tabulated. To calculate the solvent accessible surface, each of the nine regions were selected separately and calculations were performed by the method of Connolly, using a probe radius of 1.40 A° [15], [16]. Each of the nine regions was also analyzed for the hydrophobicity value based on Kyte and Doolittle [17]. The sum total of hydrophobicity value of individual amino-acid residues was tabulated. A negative value reflects low hydrophobicity or a hydrophilic nature for the region.

## Results

### Sequence Analysis

**Figure 1:**
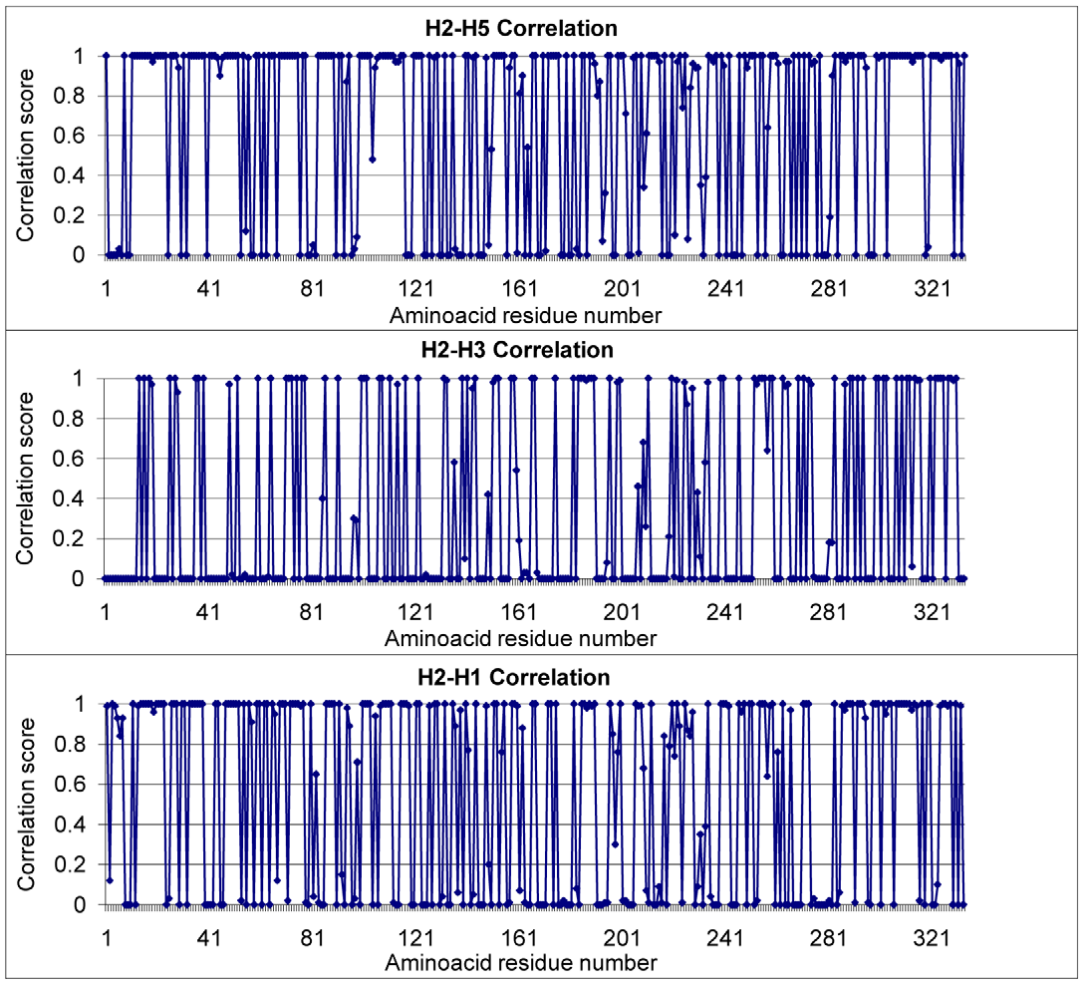
Correlation plots of Influenza A subtypes. Correlation of H2-H5, H2-H3 and H2-H1 in HA1 domain are shown. H3 residue numbering was followed in this analysis.

A comprehensive HA protein sequence analysis was performed using sequences of H1, H2, H3 and H5 subtypes of Influenza A that were available in the NCBI database, to identify all of the invariable regions. The analysis was performed in two steps; first, individual profiles of each subtype were generated and each profile was then aligned to the H2 profile to obtain the most significant alignments. The correlation plots of H2 with other subtypes reflect that subtypes H2 and H5; and H2 and H1, were clearly more similar than H2-H3 pair (Figure 1). The H2-H5 and H2-H1 pairs had correlation scores ranging from 0.8–1.0 for 66% and 56% of amino acid residues in HA1, respectively. However, a correlation score of 0.8–1.0 was observed for only 33% of the residues in the H2-H3 pair. Similar results have been reported previously by Nobusawa *et.al.* [18], who showed that H1 subtype exhibited 58.7% and 55.7% identity of the HA1 subunit to the HA1's in the H2 and H5 subtypes respectively, and least identity to the H3 subtype (35.2%).

The sequence alignment data were distributed into two components: residues with a conservation score of 0.9–1.0 were considered as conserved residues and regions containing 6 residues or longer, where more than 50% residues had conservation score of 0.9–1.0 were grouped as conserved regions. Using these criteria, we expected to miss out some important single amino-acid positions and conformational epitopes but identify regions suitable for anti-viral/peptide therapies. This data clearly identified the nine most conserved regions within all subtypes as shown in Table 1 and 2. The conservation score of each variable residue within the identified conserved region was depicted within brackets, if the variation resulted from similar residue that would lead to no change in charge/hydrophobicity. The final conservation at each position in the selected conserved regions was higher than it appears in Table 1 and 2, as the variation in the selected regions, between subtypes often resulted in similar substitutions.

**Table 1:** Identified Influenza A conserved regions/sites 1–5.

H2		H1		H3		H5			
Column Number	Consensus Residue	Column Number	Consensus Residue	Column Number	Consensus Residue	Column Number	Consensus Residue	Residue Numbering from 1HGJ	Final conservation Score
**SITE 1**									
31	(I)	32	(I)	39	(L)	29	(I)	13	(0)
32	C	33	C	40	C	30	C	14	1
33	(I)	34	(I)	41	(L)	31	(I)	15	(0)
34	G	35	G	42	G	32	G	16	1
35	Y	36	Y	43	H	33	Y	17	0
36	H	37	H	44	H	34	H	18	1
37	A	38	A	45	A	35	A	19	0.96
**SITE 2**									
89	(L)	90	(I)	96	(L)	87	(L)	70	(0.02)
90	L	91	L	97	L	88	L	71	1
91	G	92	G	98	G	89	G	72	1
92	N	93	N	99	D	90	N	73	0
93	P	94	P	100	P	91	P	74	1
94	E	95	E	101	Q	92	M	75	0
95	C	96	C	102	C	93	C	76	1
96	(D)	97	(E)	103	(D)	94	(D)	77	(0.01)
**SITE 3**									
118	C	119	C	123	C	116	C	97	1
119	Y	120	Y	124	Y	117	Y	98	1
120	P	121	P	125	P	118	P	99	1
121	G	122	G	126	Y	119	G	100	0
122	S	123	Y	127	D	120	S	101	0
123	(F)	124	(F)	128	(V)	121	(F)	102	(0)
124	N	125	A	129	P	122	N	103	0
125	D	126	D	130	D	123	D	104	0.99
126	Y	127	Y	131	Y	124	Y	105	1
127	E	128	E	132	A	125	E	106	0
128	E	129	E	133	S	126	E	107	0
129	L	130	L	134	L	127	L	108	1
130	(K)	131	(R)	135	(R)	128	(K)	109	(0)
**SITE 4**									
200	L	203	L	203	L	199	L	177	1
201	I	204	V	204	Y	200	V	178	0
202	(I)	205	(L)	205	(I)	201	(L)	179	(0)
203	W	206	W	206	W	202	W	180	1
204	G	207	G	207	G	203	G	181	1
205	(V)	208	(V)	208	(V)	204	(I)	182	(0)
206	H	209	H	209	H	205	H	183	1
207	H	210	H	210	H	206	H	184	0.99
208	P	211	P	211	P	207	P	185	0.96
**SITE 5**									
238	P	241	P	241	P	237	P	215	1
239	E	242	E	242	N	238	K	216	0.01
240	I	243	I	243	I	239	I	217	0.97
241	A	244	A	244	G	240	A	218	0
242	T	245	K	245	S	241	T	219	0
243	R	246	R	246	R	242	R	220	0.98
244	P	247	P	247	P	243	S	221	0.08
245	(K)	248	(K)	248	(R)	244	(K)	222	(0)
246	V	249	V	249	V	245	V	223	0.95

**Table 2:** Identified Influenza A conserved regions/sites 6–9.

H2		H1		H3		H5			
Column Number	Consensus Residue	Column Number	Consensus Residue	Column Number	Consensus Residue	Column Number	Consensus Residue	Residue Numbering from 1HGJ	Final conservation Score
**SITE 6**									
272	G	275	G	275	G	271	G	249	1
273	N	276	N	276	N	272	N	250	1
274	(L)	277	(L)	277	(L)	273	(F)	251	(0)
275	(I)	278	(I)	278	(I)	274	(I)	252	(0.64)
276	A	279	A	279	A	275	A	253	0.99
277	P	280	P	280	P	276	P	254	1
**SITE 7**									
304	K	307	K	306	E	303	K	280	0
305	C	308	C	307	C	304	C	281	0.97
306	Q	309	Q	308	I	305	Q	282	0
307	T	310	T	309	T	306	T	283	1
308	P	311	P	310	P	307	P	284	1
309	L	312	Q	311	N	308	M	285	0
310	G	313	G	312	G	309	G	286	1
311	A	314	A	313	S	310	A	287	0
312	I	315	I	314	I	311	I	288	1
**SITE 8**									
317	P	320	P	319	P	316	P	293	1
318	F	321	F	320	F	317	F	294	0.99
319	H	322	Q	321	Q	318	H	295	0
320	N	323	N	322	N	319	N	296	1
321	(V)	324	(V)	323	(V)	320	(I)	297	(0)
322	H	325	H	324	N	321	H	298	0
323	P	326	P	325	R	322	P	299	0
324	(L)	327	(V)	326	(I)	323	(L)	300	(0)
325	T	328	T	327	T	324	T	301	1
326	I	329	I	328	Y	325	I	302	0
327	G	330	G	329	G	326	G	303	1
328	E	331	E	330	A	327	E	304	0
329	C	332	C	331	C	328	C	305	1
330	P	333	P	332	P	329	P	306	1
331	(K)	334	(K)	333	(R)	330	(K)	307	(0.06)
332	Y	335	Y	334	Y	331	Y	308	1
333	V	336	V	335	V	332	V	309	0.99
334	(K)	337	(R)	336	(K)	333	(K)	310	(0.02)
**SITE 9**									
341	(A)	344	(V)	343	(A)	340	(A)	317	(0.1)
342	T	345	T	344	T	341	T	318	0.98
343	G	346	G	345	G	342	G	319	1
344	(L)	347	(L)	346	(M)	343	(L)	320	(0)
345	R	348	R	347	R	344	R	321	0.99
346	N	349	N	348	N	345	N	322	1
347	V	350	I	349	V	346	S	323	0
348	P	351	P	350	P	347	P	324	1

We concluded that, despite the large differences in the subtypes, there were regions of low variability that could be prime targets for anti-peptide/anti-viral responses.

### Structure Analysis

Targeting of anti-viral agents/antibodies to specific regions of the protein is complex and incompletely understood. Currently, there is no effective method of predicting the epitope structure of the pathogen and directing the antibody response to pre-defined regions using protein as an antigen. However, there is evidence that short peptides with pre-determined sequence specificity can be used to raise anti-peptide antibodies, which recognize peptide-specific region of the protein [19], [20] and monoclonal antibodies raised against synthetic peptides can cross-react with the intact protein molecule [21]. A vast majority of the literature suggests that not all regions of protein cross react with synthetic peptide antibodies and a number of structural parameters like surface exposure [22], [23], hydrophilicity and residue type [17], influence this propensity. Therefore, the above parameters were evaluated at each of the nine identified sites to predict their accessibility to anti-peptide antibodies.

The structure of HA of influenza A virus (subtype H3) strain A/Aichi/2/68 was determined by Wilson et al., 1981 [24] and, subsequently, studies of variant viruses enabled mapping of antigenic sites on the protein (reviewed in ref. 10). More recently, structures of the influenza A virus HA for three additional subtypes were solved: H1 [25], [26], H5 [27] and H9 [28]. We chose the H3 structure (PDB: 1HGJ) for our analysis to adhere to the numbering used in sequence analysis.

**Figure 2:**
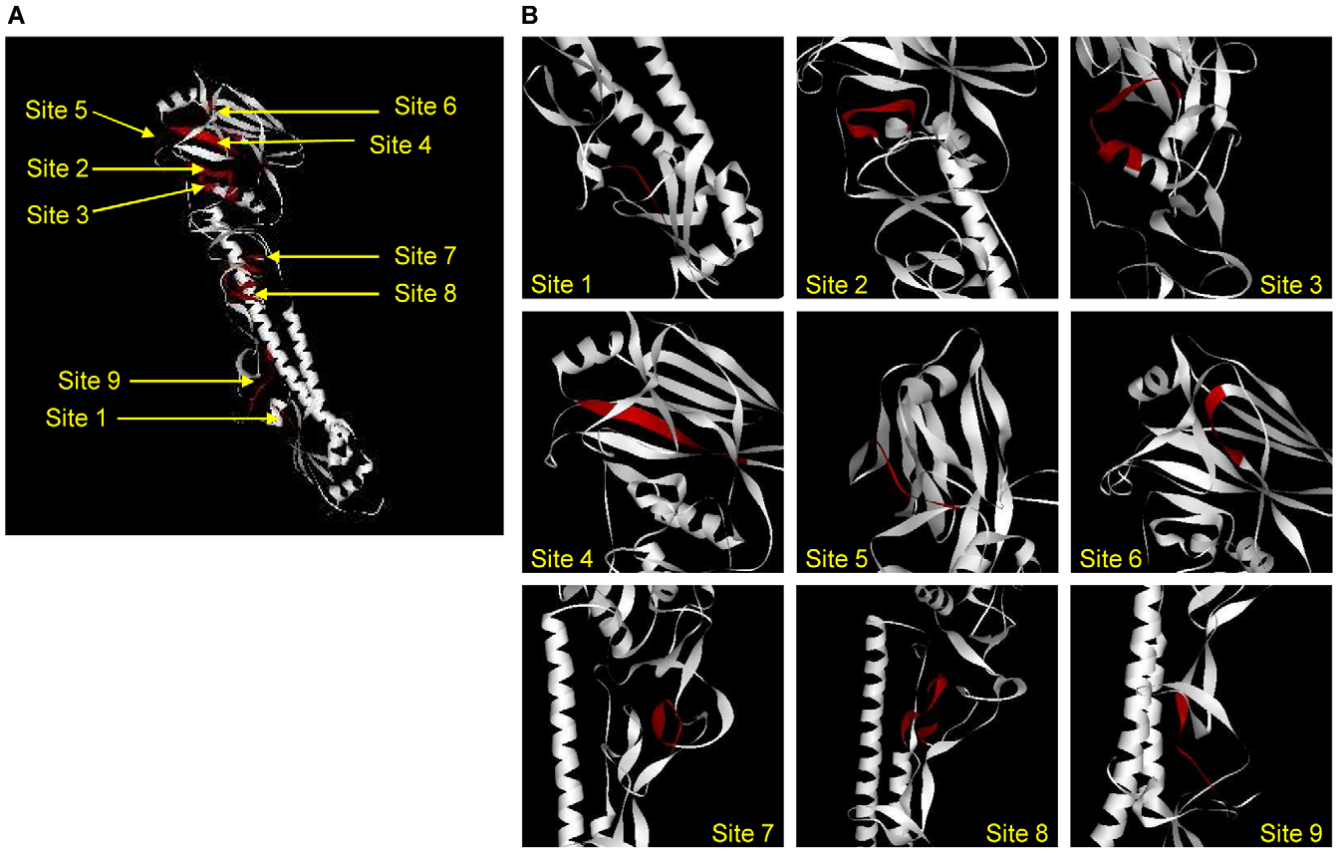
Conserved regions/sites mapped on the HA structure. (**A**) Position of the conserved regions were depicted on the HA monomer, shown as a flat ribbon model. The crystal structure of H3 subtype (PDB: 1HGJ) was used to map the identified conserved regions (colored in red). (**B**) The conserved regions shown in Figure 2A were enlarged to depict the 3-D structure details.

The position of nine identified regions were mapped on the 3-D structure of the HA monomer and shown in Figure 2A as a ribbon model. Each identified region was also enlarged to depict the details of the 3-D structure (Figure 2B). All of the identified sites 1–9 were colored in red (Figure 2B) and their secondary structure details, hydrophobicity value and solvent-accessible surface area were tabulated (Table 3). Site 1 was present at the N-terminus of HA1. This site contains the conserved cysteine residue that forms a disulphide linkage with HA2 [10]. The site exists as a loop on the HA monomer with a large solvent-accessible surface area. Site 2 and site 7 had few residues that formed a part of known antibody binding sites E and C respectively [10]. Site 2 was mostly α-helical while site 7 had mostly a β-structure. Both these sites had small solvent-accessible surface areas and may be inaccessible/buried in the monomer.

**Table 3:** Structural characteristics of conserved regions/sites.

Site no.	Rank	Total no. of residues	No. of residues and theirsecondary structure			SAS (A^2^)	SAS per residue (A^2^)	Hydrophobicity (Total)
			α-helical	β-structure	Turn/coil/loop			
1	6	7			7 (7)	554.40 (571.97)	79.2 (81.71)	5.1 (8.4)
2	5	8	5 (5)		3 (3)	186.38 (204)	26.6 (25.50)	−2.1 (−2.4)
3	4	13	5 (5)	2	6 (8)	545.95 (596.23)	41.99 (45.86)	−7.1 (−9.5)
4	8	9		8 (8)	1 (1)	105.47 (105.28)	11.71 (11.69)	2.2 (7)
5	1	9			9 (9)	554.36 (602.84)	61.59 (66.98)	−4.6 (−8.1)
6	9	6		5 (4)	1 (2)	37.20 (31.86)	6.2 (5.31)	4.6 (4.6)
7	7	9		2 (4)	7 (5)	200.73 (271.43)	22.30 (30.16)	1 (2.5)
8	3	18		6 (6)	12 (12)	972.69 (991.75)	54.04 (55.09)	−9.1 (−10.7)
9	2	8		1 (1)	7 (7)	445.79 (524.13)	55.72 (65.53)	−2.8 (1.8)

The secondary structure details, solvent accessible surface (SAS) and hydrophobicity value of each identified conserved region is shown. All the values were obtained from the H3 structure except the values in bracket that were calculated from the H1 structure.

Site 3 was composed of both, a α-helical region and a loop with a large solvent-accessible surface area, similar to site 1. This region was highly hydrophilic and has been shown to be immunodominant in eliciting antibodies against a synthetic peptide [29], [30]. However, this region does not form a part of known antibody binding site indicating that in solution, this region may acquire some conformation that is accessible only to peptide antibodies.

Sites 4 and 6 were mainly present as a β-sheet and appeared to be hidden in the monomer. Site 8 and site 9 were both found at the C-terminus of HA1 and were mainly loops with a large solvent-accessible surface area and high hydrophilicity. Site 9 has also been shown to elicit neutralizing antibody response against multiple HA subtypes [31].

This work did not identify the receptor binding site separately, but some of the conserved binding site residues were located within sites 3, 4 and 5. This lack of specific identification could be because the receptor binding site is comprised of residues that are not adjacent in the protein 3-D structure and several of the residues are not conserved [18].

A broadly cross-reactive antibody response is more probable, if the nine identified sites have adopted similar conformational structures in H1, H2 and H5 subtypes, as those in H3 subtype. In order to test this, we evaluated the structure of identified sites using the H1 structure (PDB: 1RVZ). We chose H1 structure alone due to high degree of sequence similarity between H1, H2 and H5 subtypes. In addition to sequence similarity, the nine sites had a high degree of structure conservation, with similar solvent-accessible surface and secondary structure (Table 3). Some small differences in the secondary structure were seen at sites 3, 6, and 7. The hydrophobic/hydrophilic nature of sites also remained unchanged except for site 9 which had increased hydrophobicity in the H1 structure. To identify sites that had a high potential of generating an anti-peptide response, the sites were ranked based on their solvent accessible surface and hydrophobicity. Hydrophilic sites were selected and ranked first according to their solvent accessible surface, followed by the hydrophobic sites (Table 3).

Five (1, 3, 5, 8, 9) out of the nine identified invariable regions had a high level of structure conservation between subtypes, suitable secondary structure and a significantly large solvent-accessible surface to target an anti-peptide/anti-viral response.

## Discussion

The hemagglutinin protein of influenza A virus is the major surface antigen against which neutralizing antibodies are produced and, hence, a major constituent of all vaccine formulations. However, this protein undergoes rapid evolutionary variation that leads to a change in its antigenic structure, and vaccines have to be reformulated so as to include the hemagglutinin protein from the emerging new viral strain. Thus, there is critical need for the development of an anti-viral measure to protect against any emerging Influenza A pathogen. One possible solution is an anti-peptide antibody that is directed against the conserved region of the hemaggutinin protein that is represented in all the current and possibly future viral strains. Such an anti-viral response will also dramatically speed up development of vaccines against new viral strains until viral-specific reagents are developed.

T-cells, which mediate cellular immune responses, can target more conserved peptides from internal proteins and have the potential to provide wider protection. The role of both CD4+ T-cell and CD8+ T-cell in influenza infection have been extensively studied [32], [33]. CD4+ T-cells and CD8+ T-cells recognize antigens presented by MHC class II and class I molecules, respectively. Most of the conserved internal protein sequences belong to class 1 epitopes and CD8+ response, in particular, is being considered in novel vaccine therapies [33], [34]. However, unlike antibody response, T-cell response is not sterilizing and cannot prevent infection of the host cells. Therefore, antibody based vaccine approaches, although currently strain specific, are the primary means of resistance and recovery from influenza infection.

We conducted a comprehensive HA sequence analysis of major subtypes of Influenza A virus to identify regions that were conserved between subtypes. A combination of protein sequence alignment, and the correlation score with the subtype that gave most significant alignment with other subtypes was used to identify invariant regions. This method not only gave the most significant alignment result, but also eliminated differences arising from the variable number of available sequences of different subtypes and such an approach can be useful for other viruses with high rates of mutation.

Computational searches for conserved residues have been extensively done in case of human immunodeficiency virus (HIV-1), and these results have indicated that the most highly variable regions correlate with B-cell epitopes that are responsible for viral immune escape [35]. Because it is not known how to induce or modulate the immune responses to target conserved regions, such studies have not been used for the development of vaccines/anti-viral agents. An anti-viral therapy for Influenza A virus that is based on regions that are highly conserved between all Influenza A subtypes can only have a significant strategic advantage provided that the above regions can be selectively targeted. There are many uncertainties when using whole protein as an antigen, such as localizing the antibody binding sites, directing the antibody response to certain sites and defining how amino-acid changes alter antibody binding in the context of a complex structure. The generation of anti-peptide antibodies with predefined sequence specificity is a promising alternative approach provided the antibodies that recognize and neutralize the native protein can be generated. It appears by large number of experimental reports using human immunodeficiency virus HIV-1 that many such peptides antibodies can recognize and, in some cases, neutralize some HIV-1 viruses [36], [37].

The success of binding of anti-peptide antibodies to native protein has been attributed to many structural properties and includes secondary structure, hydrophilicity and surface exposure of protein binding sites; however, the contribution of any individual parameter or a combination that can assure an antigenic response is currently unknown[22], [23], [38], [39]. Although, this structural correlation is similar to the antibody response when the whole protein is the immunogen, the immune response is not against the complex protein structure and, hence, easier to predict and control. In this context, peptides spanning the five identified conserved regions with structural parameters required for an antibody response can be tested with or without small variations for a broadly cross-reactive anti-viral response. The conserved regions adopt similar conformational structures in different subtypes, further validating the results obtained by sequence analysis and suggests that they have a potential of protecting against heterologous viral strains. This *in silico* approach will also need validation by experimental methods and in animal models.

This study establishes the identity and structural features of all conserved regions of hemagglutinin protein of Influenza A viruses. These regions/sequences can be selected as the first step in the development of new peptide vaccine strategies, and for the generation of anti-peptide antibodies as novel anti-virals and cross-reactive immunological reagents, and may provide a solution for neutralization of any existing or emerging Influenza A pathogen.

## Supporting Information

Table S1 Correlation score of H2 with other subtypes at each amino acid residue in HA1 domain. The data obtained from muscle and compass alignments was tabulated. The H3 sequence (PDB: 1HGJ) was added to follow the H3 numbering. The consensus residue at each amino acid position, the frequency of consensus residue, correlation of H2 with other subtypes and the final conservation score at each amino acid position is shown.
(0.10 MB XLS)
